# Primary cutaneous mucinous carcinoma of the scalp in a patient positive for *BRCA2* mutation

**DOI:** 10.1016/j.jdcr.2025.01.018

**Published:** 2025-02-07

**Authors:** Samer Wahood, Megan Tran, Lauryn Orsillo, Christopher DiMarco, Oliver J. Wisco, Elnaz F. Firoz

**Affiliations:** a The Warren Alpert Medical School of Brown University, Providence, Rhode Island; b College of Osteopathic Medicine of the Pacific, Northwest, Western University of Health Sciences, Lebanon, Oregon; c Department of Dermatology, The Warren Alpert Medical School of Brown University, Providence, Rhode Island; d Department of Pathology and Laboratory Medicine, The Warren Alpert Medical School of Brown University, Providence, Rhode Island

**Keywords:** *BRCA2*, breast cancer, cutaneous, mastectomy, Mohs, mucinous carcinoma, mutation, scalp

## Introduction

Primary cutaneous mucinous carcinoma (PCMC) is a rare adnexal tumor of the sweat gland with 0.07 cases per million and infrequent regional metastasis or recurrence.^1^ PCMC often presents on the eyelid or scalp and typically arises in older female patients.^2^ Histologically, PCMC is characterized by nests or strands of epithelial cells set within pools of mucin separated by thin fibrous septae.^3^ It is important to differentiate PCMC from cutaneous metastasis of mucinous adenocarcinomas from other sites given their histologic similarities.^3^ *BRCA2* mutation has been reported in some forms of mucinous carcinoma (MC) such as colorectal and gastric-type of the uterine cervix.^4^^,^^5^ We present a rare case of PCMC of the scalp in a *BRCA2*^+^ 46-year-old woman with regional metastasis.

## Case report

**Fig 1:**
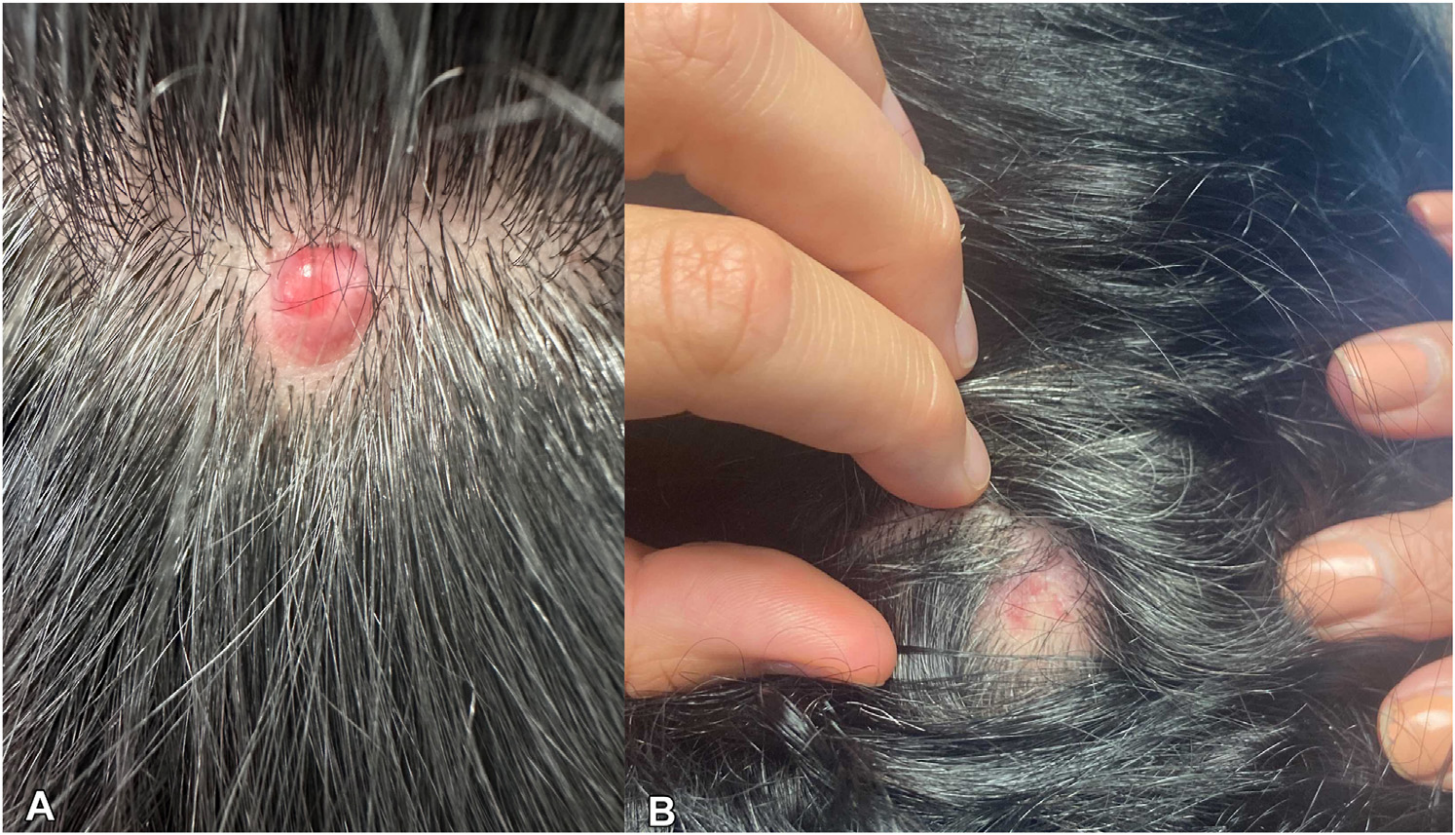
Initial and posttreatment appearance of primary cutaneous mucinous carcinoma on the right occipital scalp. Pink-to-red nodule (**A**) pretreatment and (**B**) posttreatment after Mohs micrographic surgery.

**Fig 2:**
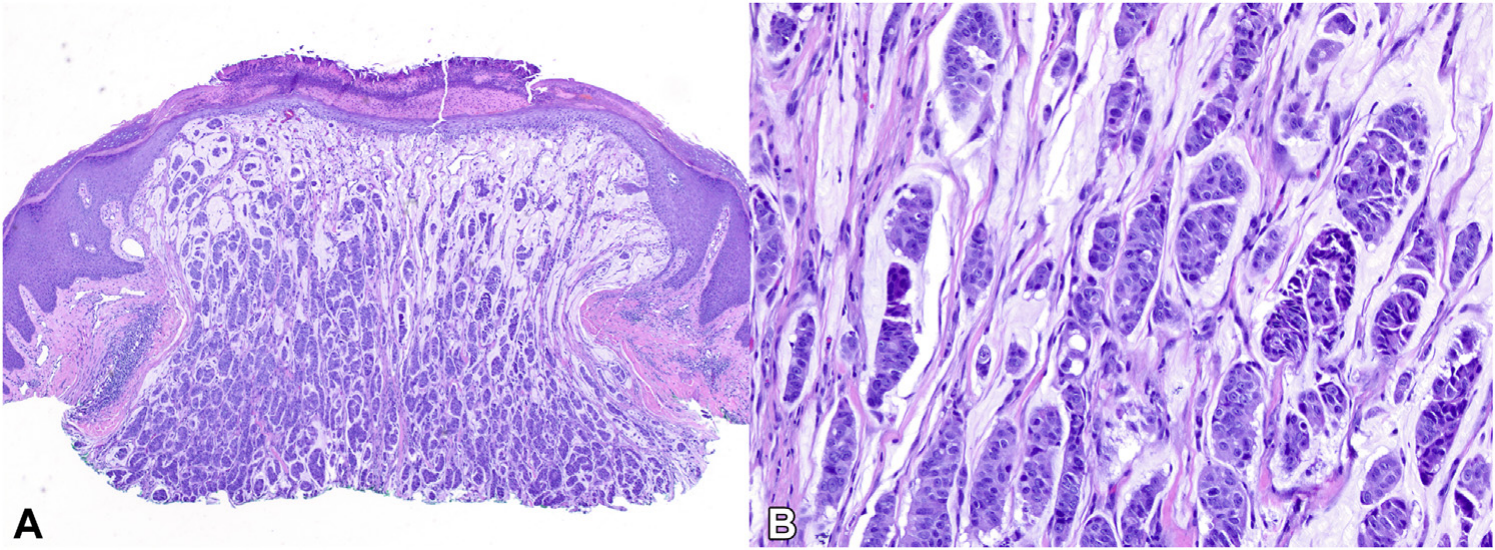
Hematoxylin and eosin (H&E) stain of primary cutaneous mucinous carcinoma. **A,** H&E shows cords and islands of epithelioid cells floating within pools of mucin. **B,** High power magnification shows pleomorphism and ductal formation. (**A** and **B,** H&E stain; original magnifications: **A,** ×40; **B,** ×200.)

**Fig 3:**
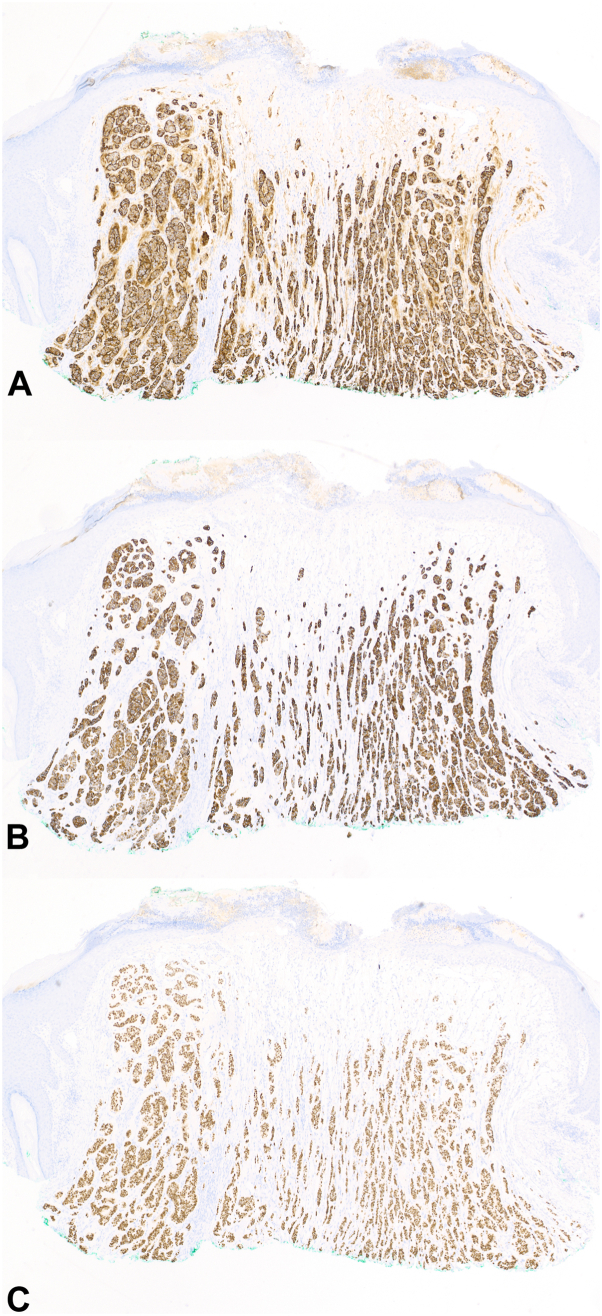
Immunohistochemical staining of malignancy. Cells are (**A**) strongly EMA^+^, (**A**) strongly CK7^+^, and (**C**) strongly ER^+^. (**A-C,** Immunohistochemical stain; original magnifications: **A-C,** ×40.)

A 46-year-old woman with medical history significant for *BRCA2* gene mutation (1222delA), estrogen receptor positive (ER^+^) ductal carcinoma in situ (DCIS) of the left breast status-post total mastectomy and breast implantation, and multiple sclerosis on natalizumab presented to her dermatologist with a new lesion on her scalp for 6 months (Fig 1, *A*). The patient’s sister and father, both known *BRCA2* carriers, paternal grandmother and aunt, and maternal grandmother and aunt share histories of breast cancer. Her father has also had lung cancer, whereas her great-paternal aunt had brain cancer. Upon examination, there was a shiny, well-demarcated 1-cm pink-to-red nodule of the right occipital scalp. No cervical, postcervical, or axillary lymphadenopathy was appreciated on examination. A shave biopsy revealed cords and islands of epithelioid cells floating within pools of mucin, pleomorphism, and ductal formation (Fig 2). The tissue was strongly positive for EMA, CK7, and ER, whereas negative for p63 and synaptophysin (Fig 3). Further staining was negative for CK5, INSM1, chromogranin, CDX-2, and Pax8. The patient underwent Mohs micrographic surgery (MMS) to remove the tumor (Fig 1, *B*).

A work-up was initiated to rule out metastatic disease, which included positron emission tomography-computed tomography (PET-CT), endoscopy, colonoscopy, and transvaginal ultrasound. No FDG avid lesions were detected. Because of the patient’s underlying multiple sclerosis, brain magnetic resonance imaging was obtained, which did not show evidence of metastatic disease or other concerning lesions. The patient was examined every 2 to 3 months without evidence of cervical or postcervical lymphadenopathy and denied any new lumps, bumps, or new symptoms.

About 1 year after diagnosis, CT scan of the head and neck showed a 1.3 cm “mildly prominent…likely reactive” left cervical lymph node that was not biopsied in the setting of 2 negative fine needle aspirations of left and right occipital nodes after neck ultrasound the same day. CT scan of the head and neck about 4 months later showed interval increase in the prominent left cervical lymph node to 1.7 cm. Image-guided core biopsy showed the same morphologic features of mucin and ductal lumina as the initial biopsy of the right occipital scalp. Immunohistochemical staining revealed the cells were >90% ER^+^, 50% progesterone receptor positive (PR^+^), HER2 equivocal (2+), GATA3^+^, and p63^−^. PET-CT showed multiple enlarged neck lymph nodes. The patient underwent bilateral neck lymphadenectomy with a plan after recovery for radiation and aromatase inhibitor therapy.

## Discussion

We present an unusual patient with known *BRCA2* mutation and history of DCIS with PCMC of the scalp treated with MMS. Despite clear margins with MMS, the tumor metastasized.

The patient’s DCIS 7 years prior was also ER^+^, raising concern for metastasis from the breast. However, the immunohistochemical profile of this MC was nonspecific for a site of origin but may have been compatible with metastasis from the breast, endometrium, or other primary site. Given the low likelihood of DCIS metastasizing and no pathologic or clinical signs of invasion of the cancer in her case, it is unlikely that the MC identified on the scalp was of breast origin. Systemic work-up did not reveal an alternate primary tumor.

Histopathology is identical for primary and metastatic cutaneous MC, and immunohistochemical staining helps differentiate PCMC from metastatic disease. A study evaluating the staining patterns of 7 cases of cutaneous MC identified CK5/6 as a marker only positive in some PCMC, not metastatic MC. The marker p63 was positive only in a few primary cutaneous and breast MC tumors. CK7 was found to be positive in all cases of PCMC, metastatic breast MC to the skin, and primary breast MC.^6^ CK5 was negative in our patient, which does not rule out primary disease. Although p63 was negative in both of our patient’s tumors, suggesting metastatic MC, systemic work-up after the patient’s DCIS and the first cutaneous MC were each initially unremarkable. GATA3 staining is sensitive for breast and urothelial carcinoma, however, a study showed it is also sensitive for cutaneous epithelial neoplasms.^7^

Being a rare tumor, PCMC lacks guidelines for management. Initial systemic work-up for patients with cutaneous MC includes a CT chest, abdomen, and pelvis and a full-body PET scan,^1^ however, the suggested follow-up imaging frequency of these scans is debatable. Cutaneous MC is histologically similar to MCs originating elsewhere in the body; therefore, CT and PET scans can aid in ruling out metastasis. A metastatic lesion was detected in our patient within about 1 year of prior PET-CT scans. Repeat CT and PET scans every 6 months may help in identifying new metastasis in time for intervention, especially in a *BRCA2*^+^ patient at higher risk of developing several types of cancers due to a germline mutation.

Our patient’s primary tumor was treated with MMS, an approach found to have the best outcomes for cure and recurrence for PCMC.^8^ A case report of ER^+^ and PR^+^ PCMC with lymph node and lung metastasis found improvement with letrozole, an aromatase inhibitor. In our case, the tumor is both ER^+^ and PR^+^, making hormonal therapy a potentially viable adjuvant therapy.^9^

Genes associated with cutaneous MC could aid in risk stratification. One study found a *GATA3* frameshift mutation (p. T418Hfs∗89) and *FOXA1* amplification in one sample of PCMC.^10^ The link between *BRCA2*, PCMC, and breast malignancy remains to be fully elucidated. Further research in PCMC is needed to better understand this uncommon tumor and whether a *BRCA2* mutation could contribute to increased PCMC metastatic potential.

## Conflicts of interest

None disclosed.
